# In vivo analysis of onset and progression of retinal degeneration in the *Nr2e3*^*rd7/rd7*^ mouse model of enhanced S-cone sensitivity syndrome

**DOI:** 10.1038/s41598-021-98271-7

**Published:** 2021-09-24

**Authors:** Giulia Venturini, Despina Kokona, Beatrice L. Steiner, Emanuele G. Bulla, Joel Jovanovic, Martin S. Zinkernagel, Pascal Escher

**Affiliations:** 1 Department of Ophthalmology, Inselspital, Bern University Hospital, Bern, Switzerland; 2 Department of BioMedical Research, University of Bern, Bern, Switzerland

**Keywords:** Disease model, Neuroscience, Development of the nervous system, Macular degeneration

## Abstract

The photoreceptor-specific nuclear receptor Nr2e3 is not expressed in *Nr2e3*^*rd7/rd7*^ mice, a mouse model of the recessively inherited retinal degeneration enhanced S-cone sensitivity syndrome (ESCS). We characterized in detail C57BL/6J *Nr2e3*^*rd7/rd7*^ mice in vivo by fundus photography, optical coherence tomography and fluorescein angiography and, post mortem, by histology and immunohistochemistry. White retinal spots and so-called ‘rosettes’ first appear at postnatal day (P) 12 in the dorsal retina and reach maximal expansion at P21. The highest density in ‘rosettes’ is observed within a region located between 100 and 350 µM from the optic nerve head. ‘Rosettes’ disappear between 9 to 12 months. Non-apoptotic cell death markers are detected during the slow photoreceptor degeneration, at a rate of an approximately 3% reduction of outer nuclear layer thickness per month, as observed from 7 to 31 months of age. In vivo analysis of *Nr2e3*^*rd7/rd7*^* Cx3cr1*^*gfp/*+^ retinas identified microglial cells within ‘rosettes’ from P21 on. Subretinal macrophages were observed in vivo and by confocal microscopy earliest in 12-months-old *Nr2e3*^*rd7/rd7*^ retinas. At P21, S-opsin expression and the number of S-opsin expressing dorsal cones was increased. The dorso-ventral M-cone gradient was present in *Nr2e3*^*rd7/rd7*^ retinas, but M-opsin expression and M-opsin expressing cones were decreased. Retinal vasculature was normal.

## Introduction

The *Nr2e3*^*rd7/rd7*^ (*rd7*: retinal degeneration 7) mouse is a mouse model of the recessively inherited enhanced short-wavelength (S)-cone sensitivity syndrome (ESCS; MIM#208100), caused by bi-allelic pathogenic variants in *NR2E3* (nuclear receptor class 2 family E member 3). The expression of the photoreceptor-specific nuclear receptor Nr2e3 is abolished in *Nr2e3*^*rd7/rd7*^ mice because of an L1 retrotransposon inserted into exon 5: the *Nr2e3* intron 5 is retained and incompletely spliced isoforms accumulate in the photoreceptor nuclei, preventing protein translation^1^. In wild-type mice, Nr2e3 expression is uniquely restricted to rod photoreceptors in the mature retina^2–4^. The rod precursors are generated over an extended developmental period, starting at embryonic day (E) 13 in the central retina, peaking around birth and ongoing until postnatal day (P) 6^5,6^. In the absence of Nr2e3, the early-born post-mitotic photoreceptor precursors that are normally committed to the rod fate become ‘blue’ S-cones^7^, yielding in a two to threefold increase in ultrastructurally normal S-cones in Nr2e3^*rd7/rd7*^ retinas^8–10^. Because the physiological function of Nr2e3 is to repress cone-specific gene expression in rods, a subset of cone genes is derepressed in Nr2e3^*rd7/rd7*^ rods^3,10,11^, resulting in a hybrid photoreceptor expressing rod and cone genes, coined ‘cod’^10^. Derepressed genes comprise those of the cone-specific phototransduction pathway^3,10,12^, and genes allowing the ‘cods’ to access the cone-specific visual cycle, as demonstrated by a markedly accelerated dark-adaptation^13^. The ultrastructure of the ‘cods’ is intermediate between normal rods and cones, with an approximately 30% larger cell body than normal rods, and an increased quantity of euchromatin and juxtanuclear mitochondria that is characteristic of cones^10^.

The autosomal recessive *rd7* phenotype was initially identified on an albino-like 77–2C2a background and then backcrossed into a pigmented C57BL/6J background^8,14–16^. At 1 month of age, Nr2e3^*rd7/rd7*^ mice showed white spots all over the retina and waves, whorls and rosette-like structures (from now on called ‘rosettes’) in the retinal outer nuclear layer^16^. At 5 months of age the white spots had nearly disappeared and waves, whorls and ‘rosettes’ had flattened out^16^. By 16 months of age the white spots were completely gone, as well as the retinal folding. The ablation of cones by mating Nr2e3^*rd7/rd7*^ mice with a mouse line carrying a transgene directing cone-specific expression of the diphtheria toxin chain A, was sufficient to abolish retinal folds and ‘rosette’ formation^17^. The hyperautofluorescent material localized within the ‘rosettes’ was shown to be due to macrophage infiltration and/or presence of microglial cells, presumably because the retinal pigment epithelium (RPE) alone is unable to remove the waste of photoreceptor outer segments within the ‘rosettes’^18,19^.

Here we analyzed in detail the progression of retinal degeneration in pigmented C57BL/6J Nr2e3^*rd7/rd7*^ mice, and discuss the findings with respect to the potential disease mechanisms underlying ESCS.

## Results

### Onset of retinal degeneration in C57BL/6J ***Nr2e3***^***rd7/rd7***^ mice

**Figure 1:**
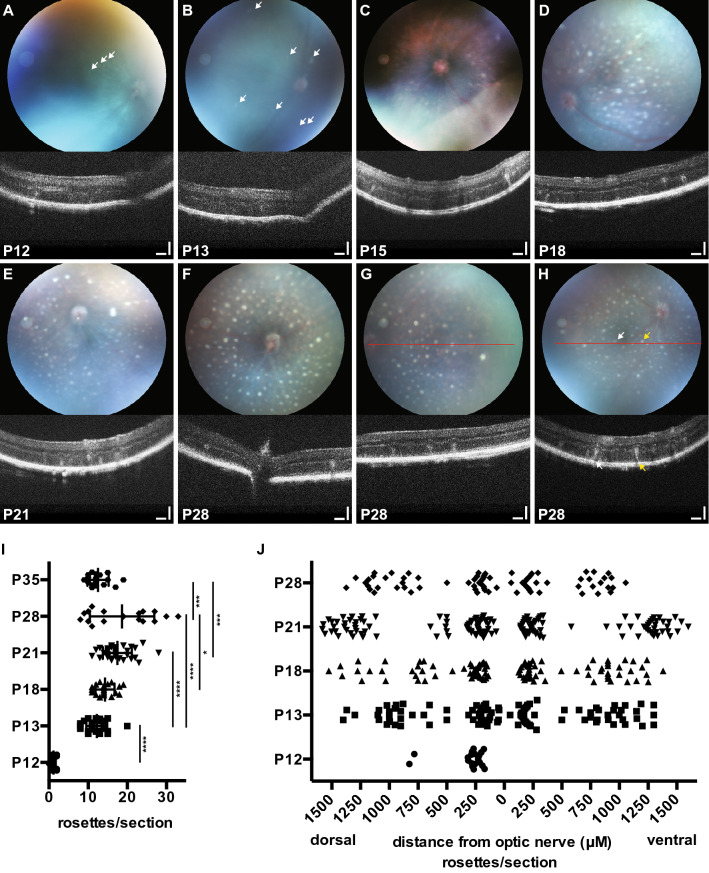
White spots and ‘rosettes’ in early postnatal C57BL/6J *Nr2e3*^*rd7/rd7*^ retinas. Fundus examination (upper panels) and representative OCT imaging (lower panels) on C57BL/6J *Nr2e3*^*rd7/rd7*^ eyes at postnatal day (P) 12 (**A**), P13 (**B**), P15 (**C**), P18 (**D**), P21 (**E**) and P28 (**F**–**H**). At P12 and P13 white spots are indicated by white arrows because retinal imaging is hampered by the developmental opacity of the lens (**A**,**B**). At P28, the region of the OCT section is indicated by a red line (**G**,**H**), and colocalization of white spots and ‘rosettes’ indicated by color-coded arrows (**H**). Scale bars: 50 µM. (**I**) Quantification of ‘rosettes’ in histological sections of C57BL/6J *Nr2e3*^*rd7/rd7*^ retinas during postnatal development. Three retinas from three different mice were analyzed. Per retina, the number of ‘rosettes’ was counted on five hematoxylin–eosin stained sections along a dorso-ventral axis containing the optic nerve head. Statistical analysis was performed by ordinary one-way ANOVA with Tukey’s multiple comparisons test. *p < 0.05 ***p < 0.001; ****p < 0.0001. (**J**) Qualitative spatio-temporal distribution of rosettes analyzed in graph I along a dorso-ventral axis relative to the optic nerve head (0).

To assess in vivo the appearance of retinal white spots and ‘rosettes’ in C57BL/6J *Nr2e3*^*rd7/rd7*^ retinas during early postnatal development, we performed fundus examination and optical coherence tomography (OCT). White spots appeared first dorsally at postnatal day 12 (P12) in C57BL/6J *Nr2e3*^*rd7/rd7*^ retinas (Fig. 1A). The number of white spots and ‘rosettes’ increased between P12 and P13 (Fig. 1B). A centrifugal expansion of white spots was then observed between P15 and P21 (Fig. 1C–E). Detailed fundus examination and OCT at P28 showed a high density of intense white spots from central to peripheral regions with peripapillary sparing (Fig. 1F), and a gradual decrease in white spots and ‘rosettes’ towards the far periphery (Fig. 1G). At this time-point we could also accurately determine that all white spots on fundus examination exactly correlated with the presence of ‘rosettes’ in the outer nuclear layer (ONL) (Fig. 1H). No ‘rosettes’ were detected at any time-point in C57BL/6J retinas (Fig. S1).

To further describe and quantify the early retinal phenotypes of C57BL/6J *Nr2e3*^*rd7/rd7*^ mice we resorted to histology (Fig. S2). At P12, the outer retina appeared wavy and ‘rosettes’ were not fully developed (Fig. S2B). At P13, ‘rosettes’ were formed and protruded towards the inner nuclear layer (INL) (Fig. S2D). The number of ‘rosettes’ increased dramatically between P12 and P13: per histological section passing through the optic nerve head, 1.2 ‘rosettes’ were observed on average at P12, and 12.2 at P13 (Fig. 1I). The average number of ‘rosettes’ increased to 14.3 at P18, 17.5 at P21 and 18.7 at P28. At P35, the average number of ‘rosettes’ had decreased to 12.5 on histological sections. We also performed a qualitative analysis of ‘rosette’ localization along a dorso-ventral axis passing through the optic nerve head (Fig. 1J). After appearance of ‘rosettes’ at P12 on the dorsal side, a pan-retinal expansion of ‘rosettes’ was already present at P13. At P18, ‘rosettes’ had expanded towards the periphery and reached maximal expansion at P21, with ‘rosettes’ observed at a distance of more than 1.5 mm from the optic disk. At all time-points and independently from the expansion towards the periphery, the highest density of ‘rosettes’ was observed in a near-central region, within a distance of 100–350 µM from the optic disk head. Typically, no ‘rosettes’ were observed in the immediate vicinity of the optic disk and in the far periphery. No ‘rosettes’ were observed at P0, P3, P5, P8 and P11 in C57BL/6J *Nr2e3*^*rd7/rd7*^ mice, and in any C57BL/6J and heterozygous C57BL/6J *Nr2e3*^*rd7/*+^ mice analyzed at these time-points (data not shown).

### Microglia are the prominent inflammatory cells in young C57BL/6J *Nr2e3*^*rd7/rd7*^ retinas

**Figure 2:**
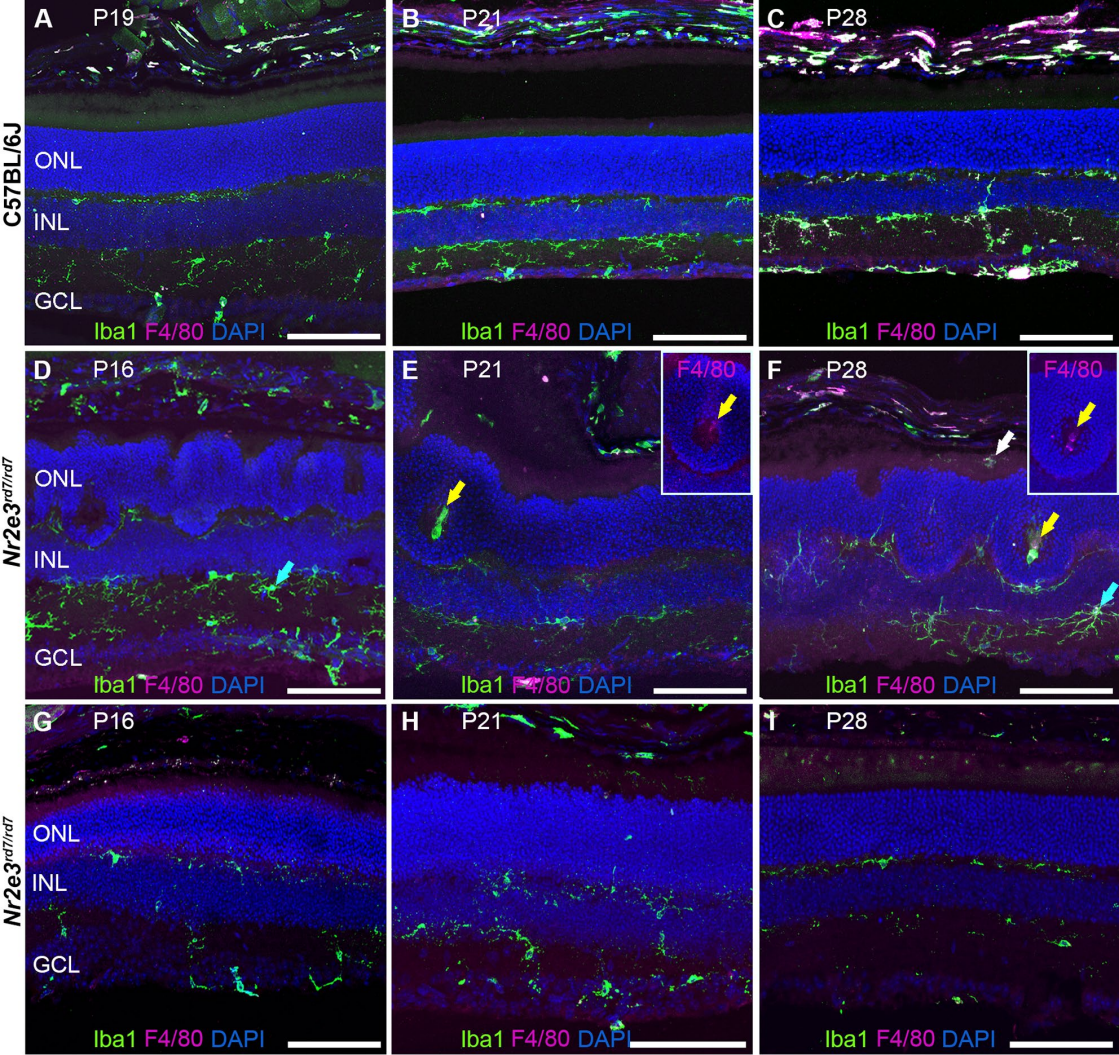
Presence of Iba1 expressing cells inside the rosettes in early postnatal C57BL/6J *Nr2e3*^*rd7/rd7*^ retinas. Iba1 and F4/80 antigen staining on C57BL/6J retinas at P19 (**A**), P21 (**B**) and P28 (**C**), and on C57BL/6J *Nr2e3*^*rd7/rd7*^ retinas at P16 (**D**,**G**), P21 (**E**,**H**) and P28 (**F**,**I**). All sections are counterstained with DAPI to visualize cell nuclei. In C57BL/6J *Nr2e3*^*rd7/rd7*^ retinas at P21 (**E**), Iba1 expressing cells accumulate inside ‘rosettes’ (yellow arrow), where F4/80 expression is faint (insert, yellow arrow). At P28 (**F**), F4/80 expressing cells are present in the subretinal space (white arrow), and a speckle-like F4/80 staining observed inside ‘rosettes’. Activated microglial cells (blue arrows) are present in the inner plexiform layer from P16 (**D**) to P28 (**F**), but not in peripheral regions devoid of ‘rosettes’ (**G**,**H**,**I**). ONL: outer nuclear layer; INL: inner nuclear layer. GCL: ganglion cell layer. Scale bars: 100 µM.

We performed immunohistochemistry on C57BL/6J retinas at P19, P21 and P28, and on C57BL/6J *Nr2e3*^*rd7/rd7*^ retinas at P16, P21 and P28 to detect the microglial cell marker Iba1 (ionized calcium-binding adapter molecule 1) and the F4/80 antigen preferentially expressed by murine mononuclear blood cells and macrophages (Fig. 2). In wild-type retinas, Iba1-positive (Iba1^+^) microglia was localized to the inner and outer plexiform layers, as well as the nerve fiber layer (Fig. 2A–C). F4/80-positive (F4/80^+^) monocytes/macrophages were present in retinal and choroidal vessels. In C57BL/6J *Nr2e3*^*rd7/rd7*^ retinas, Iba1^+^ microglia exhibited an ‘activated’ shape at P16, identified by larger cell bodies and thicker processes (Fig. 2D). At P21 and P28, a strong Iba1 immunoreactivity was detected within ‘rosettes’, but only a faint F4/80 immunoreactivity (Fig. 2E,F). Iba1^+^/F4/80^+^ cells were also detected in the subretinal space of C57BL/6J *Nr2e3*^*rd7/rd7*^ retinas at P28, suggestive of an infiltration of the monocytes/macrophages from the choroid through the RPE towards the ‘rosettes’ in the ONL at this time-point. Of note, ‘activated’ microglia was not observed in peripheral regions of P16, P21 and P28 C57BL/6J *Nr2e3*^*rd7/rd7*^ retinas, where no ‘rosettes’ were present (Fig. 2G–I).

**Figure 3:**
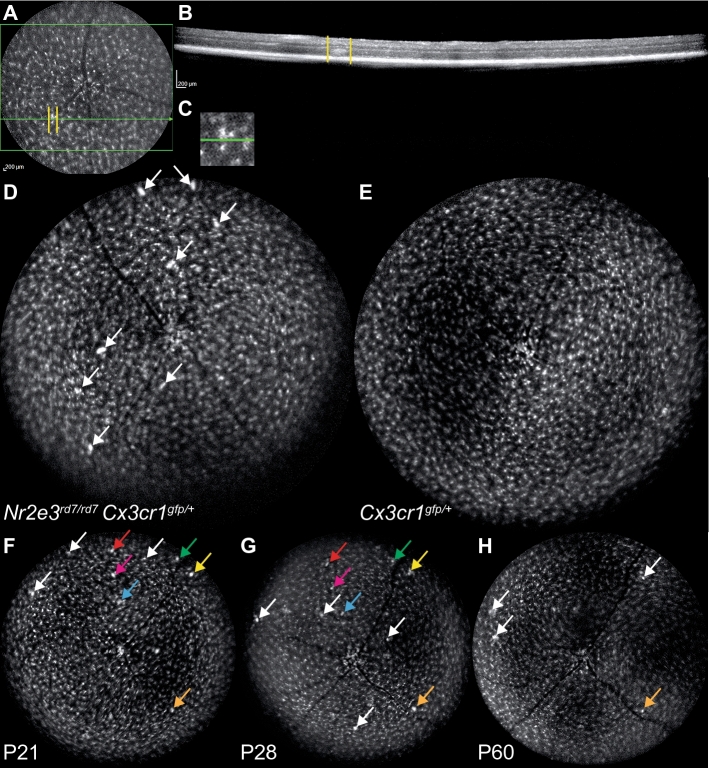
In vivo imaging of Iba1 expressing cells in *Cx3cr1*^*gfp/*+^ and *Nr2e3*^*rd7/rd7*^*Cx3cr1*^*gfp/*+^ retinas. Fundus autofluorescence imaging of *Nr2e3*^*rd7/rd7*^* Cx3cr1*^*gfp/*+^ retinas identify GFP-positive cells as a white signal (**A**). Hyperautofluorescent signals colocalize with ‘rosettes’ on OCT B-scans (**B**; scan indicated by green line and ‘rosette’ delimitated by yellow bars) and magnification shows accumulation of individual hyperautofluorescent cells (**C**). Multiple hyperautofluorescent signals are detected at P28 by fundus autofluorescence imaging of *Nr2e3*^*rd7/rd7*^* Cx3cr1*^*gfp/*+^ retinas (**D**; white arrows), but not *Cx3cr1*^*gfp/*+^ retinas (**E**). Longitudinal in vivo analysis of hyperautofluorescent signals in *Nr2e3*^*rd7/rd7*^* Cx3cr1*^*gfp/*+^ retinas at P21 (**F**), P28 (**G**) and P60 (**H**). Unique signals are indicated by white arrows, repeatedly detected signals by color-coded arrows.

To analyze retinal microglia in vivo, we crossed C57BL/6J *Nr2e3*^*rd7/rd7*^ mice with heterozygous Balb/c *Cx3cr1*^*gfp/*+^ ‘knock-in’ mice, that express selectively the green fluorescent protein (GFP) in microglial cells under the control of the endogenous *Cx3cr1* locus. We observed a decreased overall number of ‘rosettes’ in this *Nr2e3*^*rd7/rd7*^* Cx3cr1*^*gfp/*+^ mouse line of mixed genetic background in comparison to the C57BL/6J background (data not shown). Fundus fluorescence imaging readily detected GFP^+^ retinal microglial cells (Fig. 3A). By OCT we could colocalize GFP^+^ microglial cells and ‘rosettes’ and detect there both an increased fluorescent signal and an increased density of GFP^+^ microglial cells (Fig. 3B,C). These spots of increased GFP fluorescence were observed in *Nr2e3*^*rd7/rd7*^* Cx3cr1*^*gfp/*+^ retinas (Fig. 3D), but not ‘wild-type’ *Cx3cr1*^*gfp/*+^ ones (Fig. 3E). Longitudinal analysis of GFP^+^ microglial cells in a same retina allowed to detect GFP^+^ microglia at ‘rosettes’ from P21 to P60, both in persisting and newly formed ‘rosettes’ (Fig. 3F–H).

### Mild progression of retinal degeneration in old C57BL/6J *Nr2e3*^*rd7/rd7*^ mice

**Figure 4:**
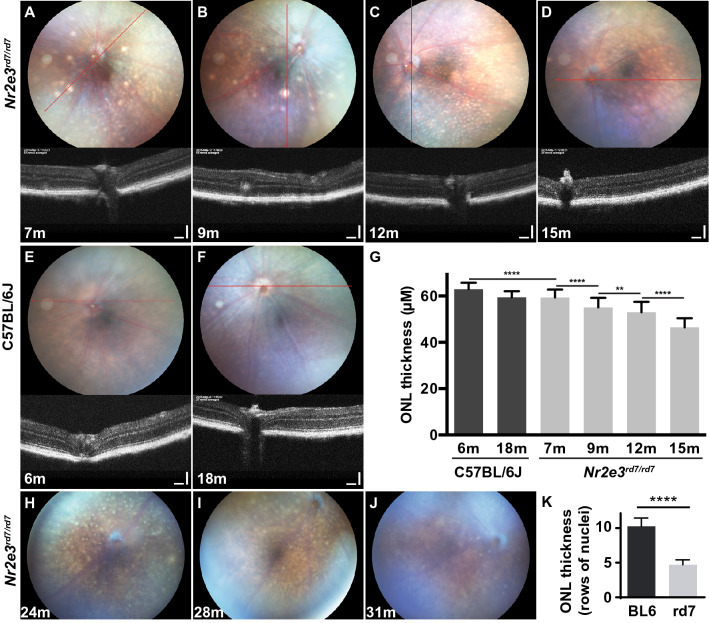
In vivo and in vitro analysis of outer nuclear layer thickness in adult C57BL/6J *Nr2e3*^*rd7/rd7*^ retinas. Fundus examination (upper panels) and OCT imaging (lower panels) on C57BL/6J *Nr2e3*^*rd7/rd7*^ retinas at 7 (**A**), 9 (**B**), 12 (**C**) and 15 (**D**) months (m), and on C57BL/6J retinas at 6 (**E**) and 18 (**F**) months. The red line on fundus images indicates the OCT scan. (**G**) Quantification of the outer nuclear layer (ONL) thickness by OCT. Average of 4 eyes from 4 different mice ± SD per time point with 7–13 ONL thickness measurements along a 2.2 cm dorso-ventral axis centered on the optic nerve head and avoiding rosette-containing regions (n = 29–87). Statistical analysis was performed by ordinary one-way ANOVA with Sidak’s multiple comparisons test. **p < 0.01; ****p < 0.0001. By performing an unpaired two-tailed t test between wild-type 6 m and 18 m a significant P value of 0.0004 is observed. Fundus photography on C57BL/6J *Nr2e3*^*rd7/rd7*^ retinas at 24 (**H**), 28 (**I**) and 31 (**J**) months (m). (**K**) Quantification of outer nuclear layer (ONL) thickness assessed by the number of rows of nuclei on hematoxylin–eosin-stained sections of retinas from 3 eyes ± SD of 30-month-old C57BL/6J (BL6) and 31-month-old C57BL/6J *Nr2e3*^*rd7/rd7*^ (rd7) mice. Five ONL thickness measurements along a dorso-ventral axis centered on the optic nerve head were done. Statistical analysis was performed by unpaired t-test. ****p < 0.0001.

Fundus photography in 7- and 9-month-old mice showed that a few hyperreflective white spots were still present in the central to mid-peripheral retina, colocalizing with ‘rosettes’ expanding to the INL on OCT (Fig. 4A,B). These bright white spots on fundus had disappeared at 12 months of age, as well as ‘rosettes’ on OCT (Fig. 4C). However, we observed by fundus photography densely scattered small beige-yellow spots, that were already visible at 7 months of age and persisted to later time-points (Fig. 4A–D). These small spots were not observed in C57BL/6J retinas (Fig. 4E,F).

On these aging mice, we performed a longitudinal analysis of the ONL thickness by OCT (Fig. 4G). As a reference we used the ONL thickness of C57BL/6J mice at 6 (N = 38) and 18 months (N = 29), where a non-significant decrease by 3.95% from 62.96 ± 0.453 µM to 60.47 ± 0.4902 µM was measured. In comparison to 6-month-old wild-type mice, the ONL thickness in C57BL/6J *Nr2e3*^*rd7/rd7*^ retinas was decreased by 5.7% at 7 months of age (59.37 ± 0.3917 µM; N = 78). By 15 months of age ONL thickness was further reduced by 21.7% (46.47 ± 0.4362 µM; N = 83). Based on this 8-month period of monitoring, we could estimate the rate of ONL thinning to less than 3% per month. Of note, the inner segment/outer segment boundary was detected in C57BL/6J *Nr2e3*^*rd7/rd7*^ retinas on OCT at all time-points (Fig. 4A–D), indicating functional photoreceptors.

The beige-yellow spots were also visible at 24, 28 and 31 months in C57BL/6J *Nr2e3*^*rd7/rd7*^ retinas (Fig. 4H–J). Histological analysis of 31-month-old C57BL/6J *Nr2e3*^*rd7/rd7*^ retinas showed a thinning of the ONL down to 4.7 rows of nuclei, in comparison to 10.3 rows of nuclei present in 30-month-old C57BL/6J retinas (Fig. 4K).

### Subretinal macrophages in aged C57BL/6J *Nr2e3*^*rd7/rd7*^ retinas

**Figure 5:**
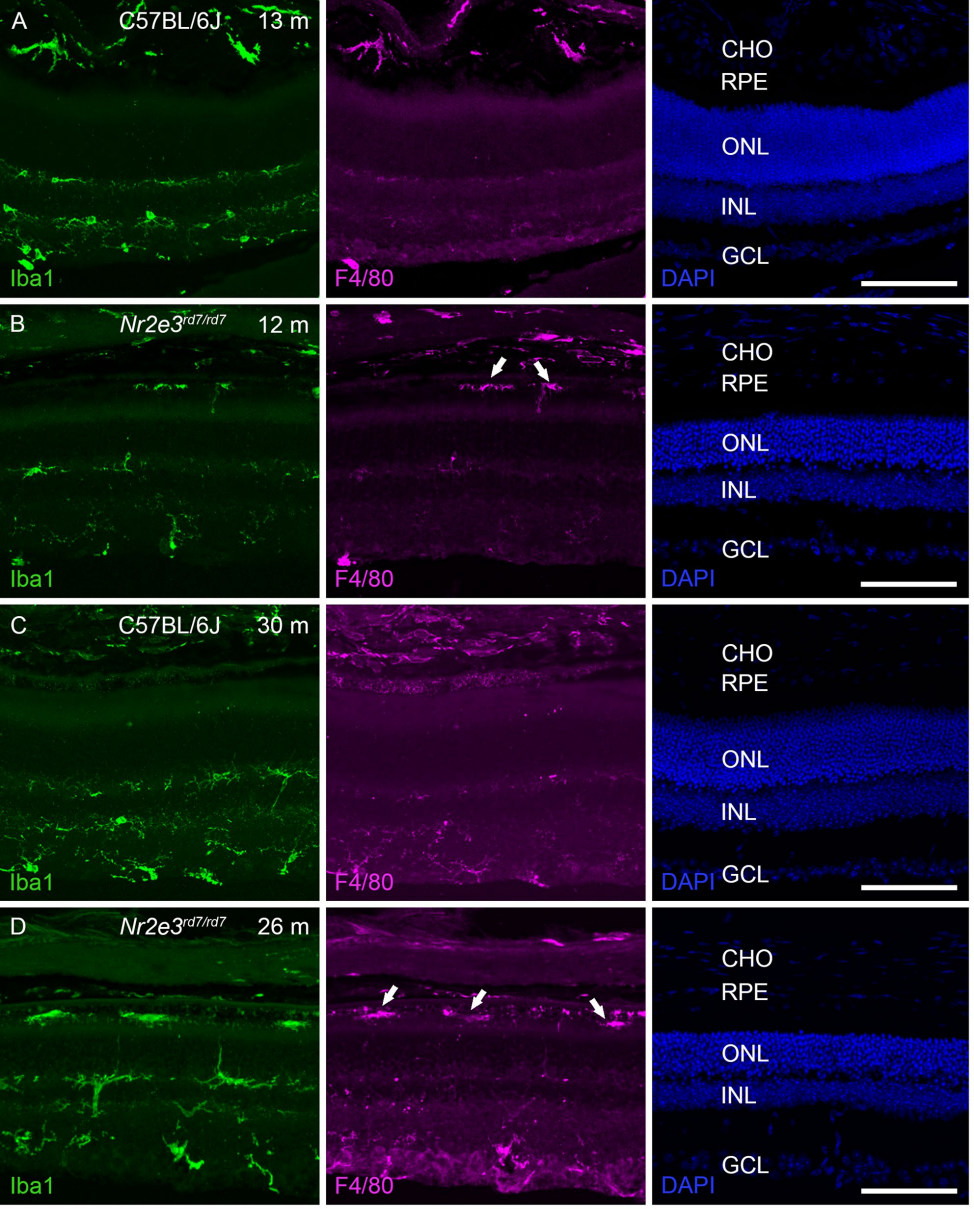
Inflammatory response in aged C57BL/6J *Nr2e3*^*rd7/rd7*^ retinas. Iba1 (left column panels) and F4/80 (middle column panels) antigen staining on C57BL/6J retinas at 13 (**A**) and 30 months (m) (**C**), and on C57BL/6J *Nr2e3*^*rd7/rd7*^ retinas at 12 (**B**) and 26 months (**D**). All sections were counterstained with DAPI to visualize cell nuclei (right column panels). Note decreased ONL thickness in C57BL/6J *Nr2e3*^*rd7/rd7*^ retinas. Localization of Iba1 positive and F4/80 positive cells in the subretinal space is indicated by white arrows in (**B**) and (**D**). CHO: choroid; RPE: retinal pigment epithelium; ONL: outer nuclear layer; INL: inner nuclear layer; GCL: ganglion cell layer. Scale bars: 100 µM.

We then performed confocal microscopy on aged C57BL/6J and C57BL/6J *Nr2e3*^*rd7/rd7*^ retinas to detect the microglial marker Iba1 and the monocyte/macrophage-selective marker F4/80 (Fig. 5). In retinas of 13- and 30-month-old C57BL/6J mice, Iba1^+^ cells were mostly detected in the inner retina, and F4/80^+^ cells were present in choroidal and retinal vessels (Fig. 5A,C). In retinas of 12-month-old C57BL/6J *Nr2e3*^*rd7/rd7*^ mice, we detected additional Iba1 expression in the subretinal space (Fig. 5B), that became more prominent in 26-month-old retinas (Fig. 5C). Importantly, this strong subretinal Iba1 expression strictly colocalized with F4/80 expression (Fig. 5B,D). Conversely, Iba1^+^ cells located in the inner retina did not express F4/80.

### Increase in non-apoptotic cell death markers in C57BL/6J *Nr2e3*^*rd7/rd7*^ retinas

**Figure 6:**
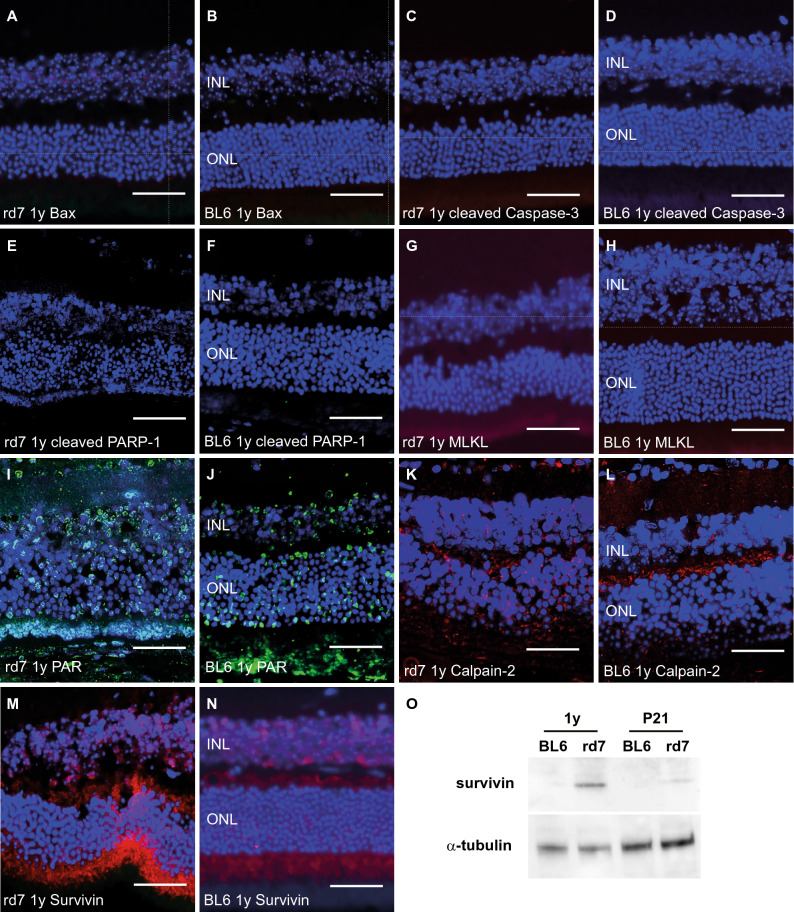
Cell death mechanisms in C57BL/6J *Nr2e3*^*rd7/rd7*^ retinas. Immunohistochemical analysis of cell death markers on 5 μM-paraffin sections of 1-year-old (1y) C57BL/6J *Nr2e3*^*rd7/rd7*^ (rd7) (**A**,**C**,**E**,**G**,**I**,**K**,**M**) and wild-type C57BL/6J (BL6) (**B**,**D**,**F**,**H**,**J**,**L**,**N**) retinas. Sections were probed with antibodies raised against Bax (**A**,**B**), cleaved Caspase-3 (**C**,**D**), cleaved PARP-1 (**E**,**F**), MLKL (**G**,**H**), PAR (**I**,**J**), Calpain-2 (**K**,**L**) and Survivin (**M**,**N**). Bax, cleaved Caspase-3, MLKL and Survivin were detected with a secondary antibody conjugated to Alexa Fluor 594 (red), cleaved PARP-1 and Calpain-2 with a secondary antibody conjugated to Cy5 (red) and PAR with a secondary antibody conjugated to FITC (green). Nuclei were stained with DAPI (blue), namely the outer nuclear layer (ONL) and the inner nuclear layer (INL). Scale bar: 50 µm. (**O**) Qualitative Western blot analysis on six pooled retinas of 21-day (P21) and 1-year-old (1y) C57BL/6J (BL6) and C57BL/6J *Nr2e3*^*rd7/rd7*^ (rd7) mice. Expression of the 16-kDa survivin and 49-kDa α-tubulin proteins were assessed.

To assess whether apoptotic or non-apoptotic cell death pathways might be involved in the slow degeneration observed in C57BL/6J *Nr2e3*^*rd7/rd7*^ retinas, we performed immunohistochemical stainings for cell death markers on paraffin sections of 1-year-old C57BL/6J and C57BL/6J *Nr2e3*^*rd7/rd7*^ retinas (Fig. 6). We did not detect immunofluorescent signals for the apoptotis regulator Bax (Fig. 6A), the pro-apoptotic cleaved Caspase 3 (Fig. 6 C) and its downstream target and DNA repair enzyme PARP-1 (poly-ADP-ribose polymerase 1) (Fig. 6E). The pro-necroptotic mixed lineage kinase domain-like protein (MLKL) was not detected by immunohistochemistry neither (Fig. 6G). In contrast, we observed increased signals for the non-apoptotic cell death marker poly(ADP-ribose) (PAR), and this predominantly on the outer side of the outer nuclear layer (Fig. 6I). Similarly, immunofluorescent signals for the non-apoptotic Calpain-2 were increased in the outer nuclear layer of C57BL/6J *Nr2e3*^*rd7/rd7*^ retinas (Fig. 6K). Finally, the expression of the inhibitor of apoptosis family member 4, also called survivin, was also markedly increased in the outer nuclear layer of C57BL/6J *Nr2e3*^*rd7/rd7*^ retinas (Fig. 6M), as well as in 1-year-old C57BL/6J *Nr2e3*^*rd7/rd7*^ retinal samples, when compared to wild-type ones (Fig. 6O).

### S- and M-opsin expression in C57BL/6J *Nr2e3*^*rd7/rd7*^ retinas

**Figure 7:**
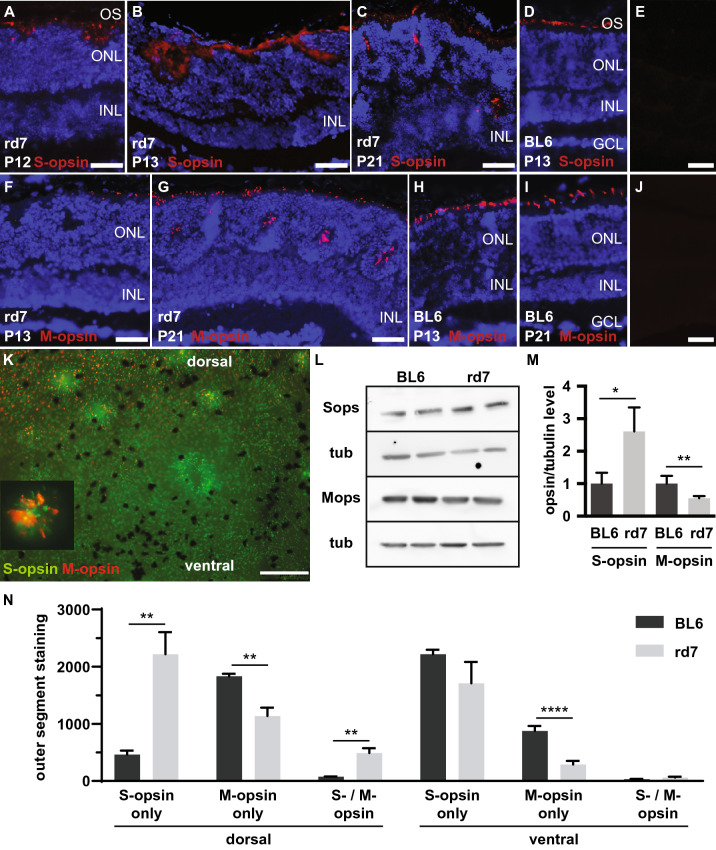
S- and M-opsin expression and distribution in early postnatal C57BL/6J *Nr2e3*^*rd7/rd7*^ retinas. S-opsin staining on C57BL/6J *Nr2e3*^*rd7/rd7*^ (rd7) retinas at P12 (**A**), P13 (**B**), P21 (**C**), and, C57BL/6J (BL6) retinas at P13 (**D**). M-opsin staining on C57BL/6J *Nr2e3*^*rd7/rd7*^ retinas at P13 (**F**) and P21 (**G**) and, C57BL/6J retinas at P13 (**H**) and P21 (**I**). All sections are counterstained with DAPI for visualization of nuclei. Negative controls with secondary antibody only for S-opsin (**E**) and M-opsin (**J**) on C57BL/6J retinas at P21. OS: photoreceptor outer segments; ONL: outer nuclear layer; INL: inner nuclear layer; GCL: ganglion cell layer. Scale bars: 25 µM. (**K**) Flat mounts of C57BL/6J *Nr2e3*^*rd7/rd7*^ retinas at P21 with co-detection of S-opsin (green) and M-opsin (red) protein expression in a central equatorial region adjacent to the optic disk. The insert shows a detailed view of rosettes containing S-opsin and M-opsin expressing cones. Scale bar: 100 µM. (**L**) Representative western blot of S-opsin (Sops: 39 kDa) and M-opsin (Mops: 40 kDa) expression in C57BL/6J (BL6) and C57BL/6J *Nr2e3*^*rd7/rd7*^ (rd7) retinas at P21. Expression of α-tubulin (tub: 49 kDa) was assessed as loading control. (**M**) Quantification of 4 independent samples ± SD assessed by Western blot to determine relative S- and M-opsin protein levels in C57BL/6J (black bars) and C57BL/6J *Nr2e3*^*rd7/rd7*^ (grey bars) retinas at P21. Statistical analysis was performed by unpaired t-test. *p < 0.05; **p < 0.01. (**N**) Quantification of cone outer segment staining for S- and M-opsin on C57BL/6J (BL6) and C57BL/6J *Nr2e3*^*rd7/rd7*^ (rd7) retinal flat mounts at P21. Three dorsal and ventral regions each were analyzed on three flat mounts of C57BL/6J retinas (n = 3) and of seven flat mounts of C57BL/6J *Nr2e3*^*rd7/rd7*^ retinas (n = 4). In C57BL/6J *Nr2e3*^*rd7/rd7*^ only regions devoid of ‘rosettes’ were analyzed. Quantifications are reported ± SEM. Statistical analysis was performed by multiple unpaired t-tests. **p < 0.01; ****p < 0.0001.

Because of the impaired photoreceptor development in *Nr2e3*^*rd7/rd7*^ retinas, we assessed in more detail S- and M-opsin expression during early postnatal development. At P12, S-opsin was detected in C57BL/6J *Nr2e3*^*rd7/rd7*^ retinas at waves starting to invaginate (Fig. 7A), and then in the fully formed ‘rosettes’ at P13 (Fig. 7B) and P21 (Fig. 7C). M-opsin was not detected in ‘rosettes’ at P13 (Fig. 7F), but at P21 all ‘rosettes’ along the dorso-ventral gradient expressed M-opsin (Fig. 7G,K). We then performed Western blot analysis on extracts from P21 retinas (Fig. 7L). Consistent with reported data at other time-points^8,9^, S-opsin expression was increased by 2.6-fold in C57BL/6J *Nr2e3*^*rd7/rd7*^ retinas in comparison to C57BL/6J levels, whereas M-opsin expression was decreased by 45% (Fig. 7M). As assessed by cone outer segment staining for S- and M-opsin on flat mounts of P21 retinas, the increase in S-opsin protein expression correlated with a 4.8-fold increase in S-opsin expressing cone outer segments in dorsal regions of C57BL/6J *Nr2e3*^*rd7/rd7*^ retinas, but not in ventral ones (Fig. 7N). We also observed an increased number of dorsally located cones expressing both S- and M-opsin. Conversely, a decrease in M-Opsin expressing outer segments was observed both in dorsal and ventral regions, by respectively 38% and 63%.

### Essentially normal vasculature in C57BL/6J *Nr2e3*^*rd7/rd7*^ mice

Finally, no vascular abnormalities were observed by fluorescein angiography in vivo in C57BL/6J *Nr2e3*^*rd7/rd7*^ retinas at 1, 3, 6, 12, 24 and 31 months of age (Fig. S3).

## Discussion

**Figure 8:**
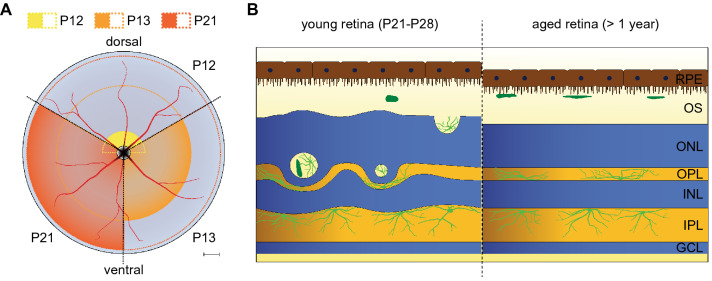
Disease mechanisms in C57BL/6J *Nr2e3*^*rd7/rd7*^ mice. Schematic drawing of disease mechanisms in the C57BL/6J *Nr2e3*^*rd7/rd7*^ mouse model of ESCS. (**A**) Early postnatal steps in ‘rosette’ formation. At P12, ‘rosettes’ appear dorsally (yellow), at P13 a pan-retinal expansion towards the periphery is observed (orange), and at P21 ‘rosettes’ have expanded to the far periphery. Scale bar: 250 µM. (**B**) Inflammatory cell population in young and aged C57BL/6J *Nr2e3*^*rd7/rd7*^ retinas. At P21 and P28 activated microglial cells (light green) migrate into the rosettes and few monocyte/macrophage-derived cells (dark green) are present in the retina. In aged retinas, the outer nuclear layer is thinning and subretinal macrophages (dark green) are regularly spaced in the subretinal space. RPE: retinal pigment epithelium; OS: outer segments; ONL: outer nuclear layer; OPL: outer plexiform layer; INL: inner nuclear layer; IPL: inner plexiform layer; GCL: ganglion cell layer.

The detailed analysis of C57BL/6J *Nr2e3*^*rd7/rd7*^ retinas identified correlations between the topographic distribution of ‘rosettes’, the maturation of photoreceptor outer segments and the density of rod photoreceptors. We detected white spots and ‘rosettes’ first at P12 on the dorsal side, then there was a centrifugal progression within a day over the entire retina reaching the most peripheral expansion at P21 (Fig. 8A). This time frame fits the central to peripheral gradient of photoreceptor maturation, where murine rod outer segments elongate at a rapid and almost linear rate from P11 to P17, and reach adult length by P19-P25^20–22^. Murine rods are very small and packed at a high overall average density of 437,000/mm^2^^23^. Quantitative analysis of photoreceptor distribution in adult C57BL/6 retinas along a 4.8 mm dorso-ventral diameter had shown an about 1.2-fold increase in photoreceptor density from the posterior pole towards a region at about 600 µM from the posterior pole, and then an about 1.4-fold decrease towards the periphery^23,24^. We observed the highest density of ‘rosettes’ within a region located within 100–350 µM from the optic nerve head along a 3.6 mm dorso-ventral diameter in young postnatal eyes, that corresponds to this region of higher photoreceptor density. In C57BL/6J *Nr2e3*^*rd7/rd7*^ retinas the dense packing of photoreceptors is exacerbated by an increase in ‘cod’ cell body size by up to 30%^10^. Additionally, the observed 4.8-fold increase in dorsal cones expressing S-opsin may further exacerbate spatial constraints and the first appearance of ‘rosettes’ in the dorsal retina at P12 related to S-cone outer segment development. Additional dorsal S-cones and photoreceptor outer segment maturation are plausible causes of the initiation of ‘rosette’ formation and expansion towards the periphery. In presence of high photoreceptor density and larger photoreceptor cell bodies spatial constraints are then further exacerbated. ‘Rosette’ formation can be regarded as an attempt to accommodate larger photoreceptor cell bodies for a same RPE surface by ‘pushing’ photoreceptors towards the periphery. In support of this spatial constraint hypothesis, the genetic removal of cones was sufficient to generate enough space in the retina and prevent ‘rosette’ formation in *Nr2e3*^*rd7/rd7*^ retinas^17^. Consistent with ‘rosette’ formation driven by rod density, patients affected by recessive *NR2E3*-linked ESCS typically exhibit pathological fundus and autofluorescence changes in the perimacular-to-mid-peripheral region^25–27^, where rods are at their highest density of 160,000/mm^2^^28^. Whether this mid-peripheral region also contains an increased number of S-cones in ESCS patients remains elusive^29^.

With respect to cone opsin expression, we postulate that the respective increase in S-opsin and decrease in M-opsin expression we assessed in *Nr2e3*^*rd7/rd7*^ retinas, reflects the reported ERG findings of enhanced S-cone function but decreased M-cone function in human patients^30,31^.

White spots and ‘rosettes’ gradually disappeared between 9 and 12 months of age in adult C57BL/6J *Nr2e3*^*rd7/rd7*^ retinas. We also determined an approximately 3% decrease per month in ONL thickness in the C57BL/6J *Nr2e3*^*rd7/rd7*^ retina between 7 and 31 months of age. This is consistent with retinal function within normal limit until 5 months of age, but reduced by 50% at 16 months as assessed by electroretinography (ERG)^16^. The slow-progressing retinal degeneration observed in C57BL/6J *Nr2e3*^*rd7/rd7*^ mice resembles to what is observed in ESCS patients^32^. Because we detected increased expression of the non-apoptotic cell death markers PAR, Calpain-2 and Survivin, but not of the pro-apoptotic Bax, cleaved Caspase 3 and cleaved PARP-1 and the pro-necroptotic MLKL markers, our data suggest that the slow retinal degeneration observed in aging C57BL/6J *Nr2e3*^*rd7/rd7*^ retinas is driven by non-apoptotic cell death pathways, similar to what is observed in a vast majority of analyzed murine hereditary retinal degeneration models^33^.

The presence of immune cells within ‘rosettes’ may mediate the waste removal of trapped photoreceptor outer segments, normally phagocytosed by the RPE^18,19^. We observed an increased fluorescence signal in vivo in microglial cells around rosettes, which would be consistent with an increased uptake of hyperautofluorescent photoreceptor outer segments. Importantly, our analyses show an early phase of microglial cell migration into ‘rosettes’, followed by the immigration of monocytes/macrophages across the RPE (Fig. 8B). The small yellow spots observed by fundus photography in retinas of old mice have been associated with subretinal microglial cells^34^, but our immunohistochemical analysis detecting subretinal localization of F4/80^+^ cells in aged C57BL/6J *Nr2e3*^*rd7/rd7*^ retinas is suggestive of subretinal macrophages. We hypothesize that the subretinal fibrosis described in ESCS may be caused by the presence of subretinal macrophages^35^. Further analyses will be necessary to establish the respective roles of infiltrated macrophages and resident activated microglia in this retinal degeneration.

Taken together, our data identified additional S-cones and photoreceptor outer segment maturation as likely triggers of ‘rosette’ formation. Initial microglia migration towards ‘rosettes’ is followed by monocyte/macrophage immigration. These findings further illustrate the validity of the *Nr2e3*^*rd7/rd7*^ mouse retina to study ESCS-associated disease mechanisms^36^.

## Methods

### Animals

All experiments performed in this study were in accordance with the ARRIVE guidelines and were approved by the Veterinary Offices of the Canton of Bern (authorization BE17/19). C57BL/6J (RCC, Basel, Switzerland) and B6.Cg-*Nr2e3*^*rd7*^/J mice (Jackson Laboratory, Bar Harbor, ME, USA) were kept in a 12-h light–dark cycle with unlimited access to food and water. In order to increase the fertility of the B6.Cg-*Nr2e3*^*rd7*^/J mice that had been backcrossed over 8 generations to homozygosity at Jackson Laboratory and had a typical litter size of 1–3 pups, we backcrossed them over 4 generations to the C57BL/6J genetic background, resulting in our C57BL/6J *Nr2e3*^*rd7/rd7*^ line with an average litter size of 6–8 pups. During backcrossing, all litters were systematically checked for the presence of the *rd1* and *rd8* mutations^37,38^. C57BL/6J *Nr2e3*^*rd7/rd7*^ mice were further crossed with homozygous mice selectively expressing green fluorescent protein (GFP) in microglia under the control of the *Cx3cr1* gene were obtained by crossbreeding wild type Balb/cAnNCrl females with male transgenic homozygous fractalkine receptor reporter mice (*Cx3cr1*^*gfp/gfp*^) on a Balb/c background^39^.

### In vivo imaging

Mice were anesthetized by intraperitoneal injection (Sterican^®^ 23 gauge needle; Braun, Melsungen, Germany) of a ketamine 6 mg/ml (Ketalar^®^; Pfizer, New York, NY, USA) and medetomidine 0.1 mg/ml (Domitor^®^; Pfizer) solution, diluted in a sterile NaCl 0.9% solution, at 10 µl/g of body weight^40^. For mydriasis, eyes were rinsed with a drop of a sterile irrigating solution (Balanced Salt Solution; Alcon, Fort Worth, TX, USA) for 10 s. Pupils were dilated with a subsequent drop of tropicamide 0.5% (SDU Faure; Novartis, Basel, Switzerland) for up to 2 min and of phenylephrine hydrochloride 100 mg/ml (Neosynephrin-POS^®^ 10%; Ursapharm, Saarbrücken, Germany) for 1 min. After rinsing, the eyes were constantly humidified with 0.3% methyl-hydroxy-propyl-cellulose and dextran solution (Tears Naturale^®^; Alcon Laboratories, Fort Worth, TX, USA). The eye to be examined was then humidified with a drop of 2% hypromellose (Methocel^®^ 2%; Omnivision). Imaging was performed either on a retinal imaging microscope for small animals (Micron III; Phenix Research Laboratories, Pleasanton, CA, USA) or a confocal laser scanning ophthalmoscope (Heidelberg Spectralis HRA2; Heidelberg Engineering GmbH, Heidelberg, Germany). After funduscopy, mice were processed for OCT and/or fluorescein angiography. For OCT, retinal scans were acquired on both eyes of all animals and centered at the optic nerve head. Outer nuclear layer measurements were performed with InSight Software. For fluorescein angiography, anesthetized mice were injected intraperitoneally with a 5% sodium fluorescein solution (Akorn, Decatur, IL, USA) at a dose of 40 µl per 6 g of body weight. Anesthesia was reversed by intraperitoneal injection of an atipamezole 0.1 mg/ml solution (Antisedan^®^; Pfizer), diluted in sterile NaCl 0.9%, at a concentration of 12 µl/g of body weight.

### Histochemistry and immunohistochemistry

For histochemical analysis, eyes were enucleated, fixed in 4% paraformaldehyde-1xPBS overnight at 4 °C, cryoprotected by immersion in 30% sucrose-1xPBS overnight at 4 °C and embedded in freezing compound (30% albumin/3% gelatin in 1xPBS). Ten-μm cryosections were collected on Superfrost^®^Plus glass slides (Menzel, Braunschweig, Germany) and dried at room temperature for at least 1 h before being stained with hematoxylin–eosin (Sigma, St. Louis, MI, USA)^41^. For immunohistochemistry, eyes were enucleated, fixed in 4% paraformaldehyde-1xPBS for 45 min at 4 °C, cryoprotected by immersion in 10% sucrose-1xPBS for 1 h, in 20% sucrose-1xPBS for 2 h and in 30% sucrose-1xPBS overnight at 4 °C, and 10-μM cryosections were collected as described above. Cryosections were hydrated with 1xPBS and blocked for 1 h in blocking solution (2% normal goat serum, 0.2% Triton X-100 in 1xPBS). Then, all antibodies were diluted in blocking solution and incubated overnight at 4 °C: a rabbit polyclonal antibody directed against human S-opsin (OPN1SW; ARP59911, Aviva Systems Biology, San Diego, CA, USA) was diluted 1/250, a rabbit polyclonal antibody against human L/M-opsin also cross reacting with mouse M-opsin (OPN1MW; AB5405, Merck Millipore, Darmstadt, Germany) was diluted 1/1000, a rabbit polyclonal antibody against Iba1 (019-19741; Wako Pure Chemical Industries Ltd., Osaka, Japan) was diluted 1/1000 and a rat monoclonal antibody against the macrophage F4/80 antigen (ab6640; Abcam, Cambridge, UK) was diluted 1/250. Sections were then washed three times for 5 min in 1xPBS. Secondary antibodies conjugated to Alexa Fluor 488 or 594 (Molecular Probes; Invitrogen, Carlsbad, CA, USA) were diluted at 1/1000 in blocking solution and incubated for 1 h at room temperature in the dark. Sections were washed 3 times for 5 min in 1xPBS and then counterstained for 10 min with 6-diamidino-2-phenylindole (DAPI) to visualize the cell nuclei. Slides were washed three times for 5 min in 1xPBS, before mounting in Citifluor (Citifluor Ltd; London, UK). Images were acquired on a Leica MZ 16F stereomicroscope (Leica, Heerbrugg, Switzerland) or a Leica DM 6000B microscope. Confocal microscopy was performed on an inverted Zeiss LSM 710 fluorescence microscope (Carl Zeiss Meditec AG, Jena, Germany) and Z-stacks of 100 µM with 5-µM intervals were acquired.

For immune-detection of proteins in cell death mechanisms, 5 μM paraffin sections of retinas on SuperFrost Plus™ adhesion slides (J1800AMNZ; Thermo Fisher Scientific, Waltham, MA, USA) were stained with rabbit polyclonal antibodies raised against Survivin (PA116836; Thermo Fisher Scientific), Bax (PA511378; Thermo Fisher Scientific), MLKL (ab172868; Abcam), and cleaved Caspase-3 (9661S; Cell Signaling, Danvers, MA, USA) diluted at 1/200 in blocking solution (5% Normal Goat Serum (X090710; Agilent Technologies, Santa Clara, CA, USA), 0.2% Triton X-100 (9002-93-1; Sigma-Aldrich, St. Louis, MO, USA) in 1X PBS pH 7.4 (70011044; Thermo Fisher Scientific). Immunodetection was done with a secondary anti-rabbit Alexa Fluor 594 antibody (A11012; Life Technologies, Carlsbad, CA, USA) diluted 1/1000 in blocking solution. Slides were mounted with VECTASHIELD^®^ HardSet™ Antifade Mounting Medium containing DAPI (H-1500; Vector Laboratories, Burlingame, CA, USA). Rabbit polyclonal antibodies raised against cleaved PARP-1 (ab32064; Abcam) and Calpain-2 (AB1625; Merck Millipore, Burlington, MA, USA) were used at 1/100 in blocking solution and immunodetection was done with a secondary anti-rabbit Cy5 antibody (A10523; Life Technologies) diluted 1/1000 in blocking solution. A mouse monoclonal antibody raised against PAR (ALX-804-220-R100; Enzo Life Sciences, Farmingdale, NY, USA) was used at 1/100 in blocking solution and immunodetection was done with a secondary anti-mouse FITC antibody (ab6785; Abcam) diluted 1/1000 in blocking solution.

### Retinal flat mount

Retinas were isolated under a dissecting microscope and gently transferred to a 96-well cell culture dish containing 4% paraformaldehyde-1xPBS for 2 h. They were washed three times for 5 min in 1xPBS and blocked for 1 h at room temperature with gentle agitation in blocking buffer (2% normal goat or horse serum, 0.2% Triton X-100 in 1xPBS). Incubation in primary antibody (OPN1SW, sc-14365, Santa Cruz, Dallas, TX, USA, 1/100 in blocking solution; OPN1MW, AB5405, Merck Millipore, Darmstadt, Germany, 1/1000 in blocking solution) was performed overnight at 4 °C with gentle agitation. Secondary antibodies, diluted 1/1000 in blocking solution, were incubated for 1 h at room temperature. Retinas were then washed 3 times in 1xPBS and transferred to a glass slide. To flatten the retinas, four cuts were made, equidistant apart, from the periphery of the retina towards the center. Slides were mounted in Citifluor before images were acquired on a Leica DM 6000B microscope. Cone outer segments were quantified using Fiji/ImageJ version 1.51 (http://imagej.nih.gov/ij; National Institutes of Health, Bethesda, Maryland USA). Measurements were done in dorsal and ventral regions on rectangles of 570 × 1370 pixels. Background was removed using in-built Otsu thresholding method with analyzed particle size (volume) set to 10–400 pixel^2^.

### Western blot

On ice, two mouse retinas were homogenized with a plastic pestle in 100 µl of a buffer containing 100 mM NaCl, 50 mM Tris pH 7.5, 1 mM EDTA, 0.1% Triton X-100, and freshly added protein inhibitors (Complete; Roche). Thirty micrograms of protein extracts were resolved on 10% SDS-PAGE gels followed by transfer on PVDF membrane (Immobilon-P; Merck Millipore). Membranes were blocked in 5% non-fat dried milk before being immunoassayed using rabbit polyclonal antibodies against survivin (diluted 1/1000; Thermo Fisher PA1-16836), S-opsin and M-opsin (diluted 1/1000; Merck Millipore) and a mouse monoclonal antibody against α-tubulin (1/5000; Sigma, Buchs, Switzerland). The secondary ECL™ donkey anti-rabbit and sheep anti-mouse IgG horseradish peroxidase-conjugated antibodies were diluted 1/15,000 (GE Healthcare, Buckinghamshire, UK). Proteins were detected by chemiluminescence using the Amersham ECL Advance Western Blotting Detection Kit (GE Healthcare) in a Fujifilm LAS-4000 mini imaging system (Bucher Biotec, Basel, Switzerland). Molecular weight markers were purchased at Fermentas (PageRuler^TM^Plus).

### Statistical analysis

All results were expressed as means ± SD or ± SEM, and the number of samples and experiments indicated in text and figure legends. Statistical analyses were performed with Prism 8.2.0 (GraphPad Software Inc., La Jolla, CA, USA).

## Supplementary Information

Supplementary Information.
