# Author Correction: In vivo analysis of onset and progression of retinal degeneration in the *Nr2e3*^*rd7/rd7*^ mouse model of enhanced S-cone sensitivity syndrome

**DOI:** 10.1038/s41598-021-00207-8

**Published:** 2021-10-13

**Authors:** Giulia Venturini, Despina Kokona, Beatrice L. Steiner, Emanuele G. Bulla, Joel Jovanovic, Martin S. Zinkernagel, Pascal Escher

**Affiliations:** 1 Department of Ophthalmology, Inselspital, Bern University Hospital, Bern, Switzerland; 2 Department of BioMedical Research, University of Bern, Bern, Switzerland

Correction to: *Scientific Reports* 10.1038/s41598-021-98271-7, published online 24 September 2021

The Acknowledgements section in the original version of this Article was incomplete.

“This work was supported by the Swiss National Science Foundation (grant 31003A_138492 to P.E.) and the Gottfried-und-Julia-Bangerter-Rhyner-Stiftung (to P.E.).”

now reads:

“This work was supported by the Swiss National Science Foundation (grants 31003A_138492 and 31003A_169237 to P.E.) and the Gottfried-und-Julia-Bangerter-Rhyner-Stiftung (to P.E.)."

The original Article has been corrected.

