# Supplementary material for: In vivo analysis of onset and progression of retinal degeneration in the Nr2e3rd7/rd7 mouse model of enhanced S-cone sensitivity syndrome
Source: Sci Rep. 2021 Sep 24;11:19032. doi: 10.1038/s41598-021-98271-7 (PMC8463594; doi:10.1038/s41598-021-98271-7)
Supplement: Supplementary file 1 — Supplementary Information. [file 41598_2021_98271_MOESM1_ESM.pdf]

Supplementary Information

In vivo analysis of onset and progression of retinal degeneration in the *Nr2e3*$^{rd7/rd7}$ mouse model of enhanced S-cone sensitivity syndrome

Giulia Venturini[1,2], Despina Kokona[1,2], Beatrice L. Steiner[1,2], Emanuele G. Bulla[1,2], Joel Jovanovic[1,2], Martin S. Zinkernagel[1,2] and Pascal Escher[1,2,*]

[1]Department of Ophthalmology, Inselspital, Bern University Hospital
[2]Department of BioMedical Research, University of Bern, Bern, Switzerland

Table of contents

Figure S1: Fundus examination and optical coherence tomography (OCT) imaging of C57BL/6J retinas.

Figure S2: Hematoxylin-eosin staining of C57BL/6J *Nr2e3*$^{rd7/rd7}$ retinas

Figure S3: Fluorescein angiography on C57BL/6J *Nr2e3*$^{rd7/rd7}$ retinas

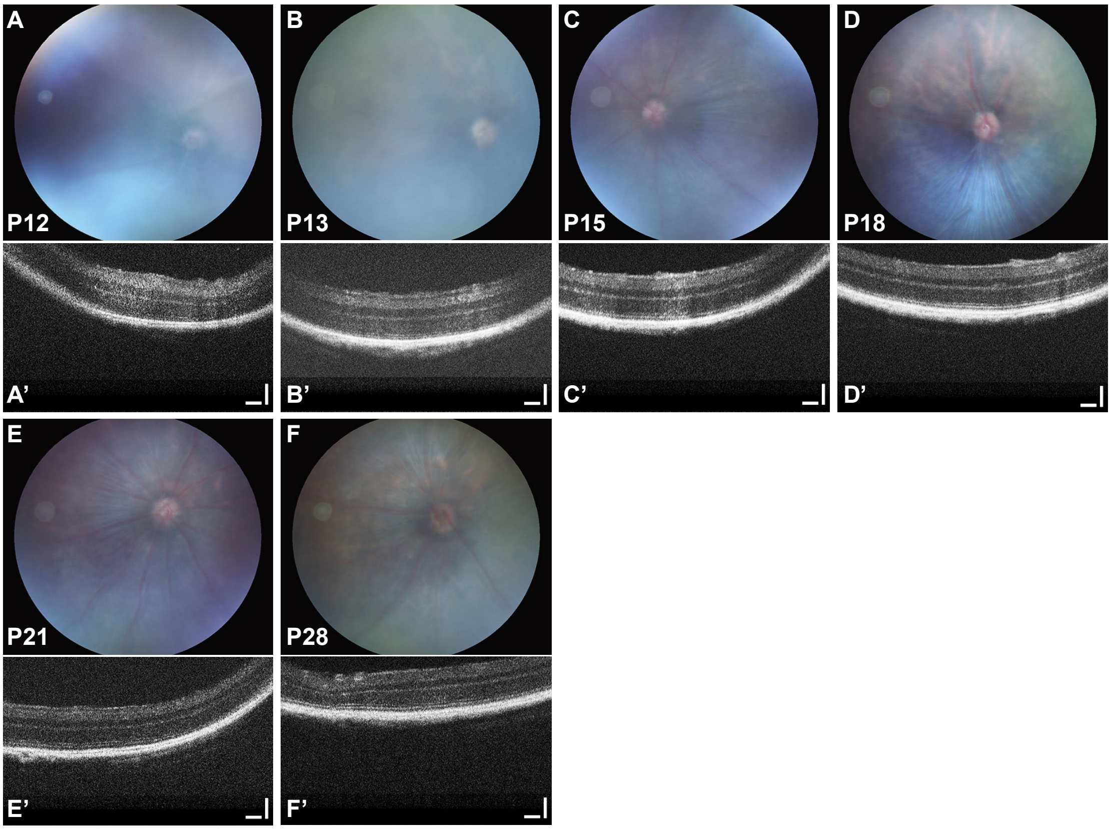

***Supplementary Figure S1*: Fundus examination and optical coherence tomography (OCT) imaging of C57BL/6J retinas.** Fundus examination (color panels) and OCT imaging (gray scale panels numbered in ') on C57BL/6J retinas at postnatal day (P) 12 (A), P13 (B), P15 (C), P18 (D), P21 (E) and P28 (F). Fundus imaging is hampered by the developmental opacity of the lens at P12 and P13. No white spots were observed at any time-point in C57BL/6J retinas. Scale bars: 50 μM.

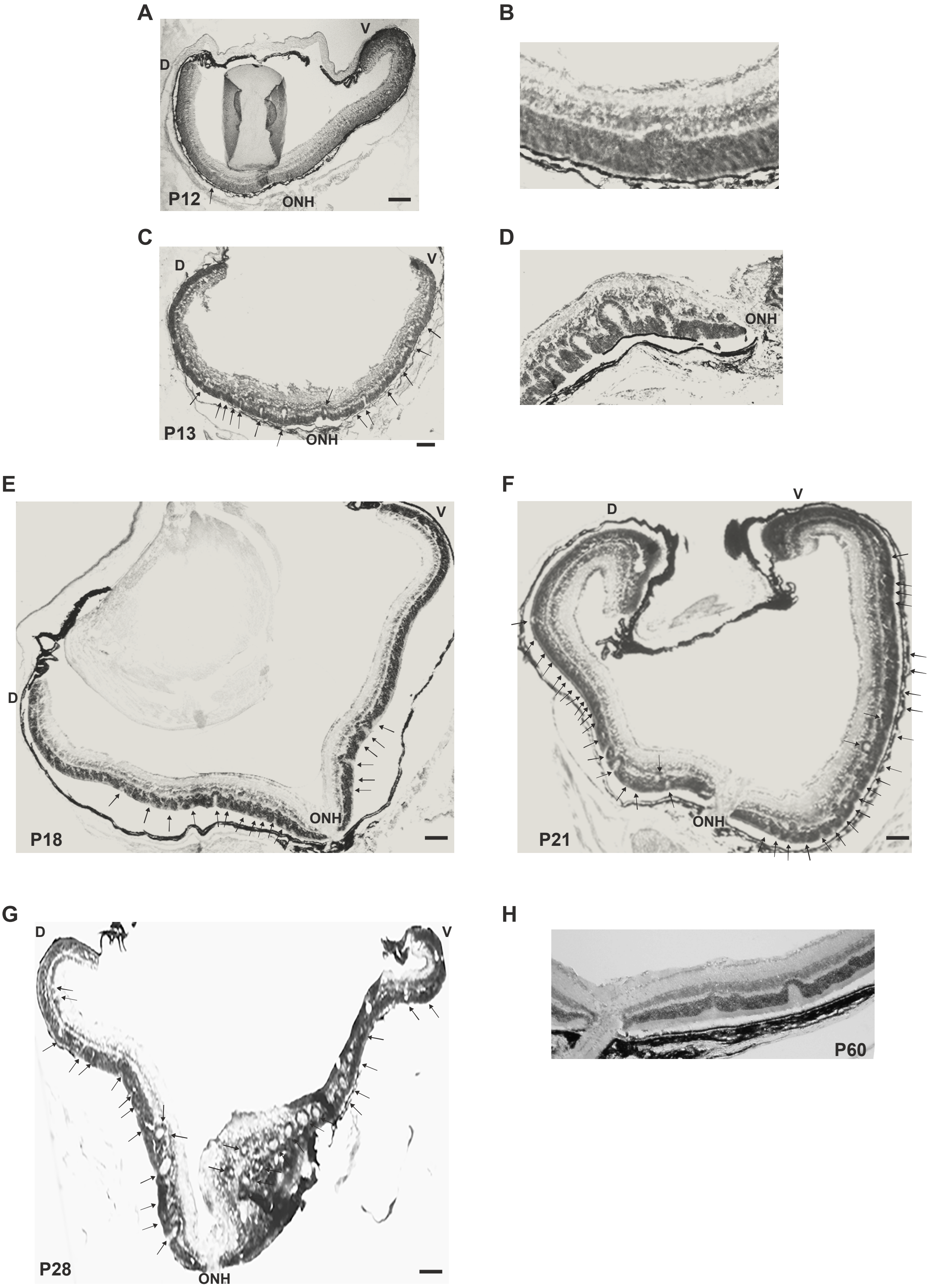

***Supplementary Figure S2*: Hematoxylin-eosin staining of C57BL/6J *Nr2e3^rd7/rd7^* retinas.** Rosette distribution was assessed on hematoxylin-eosin stained C57BL/6J *Nr2e3^rd7/rd7^* retinas along a dorso (D) - ventral (V) axis at P12 (A), P13 (C), P18 (E), P21 (F) and P28 (G). Rosettes are indicated by black arrows. Magnification of rosette formation at P12 (B) and P13 (D). At P60, rosettes persist predominantly in vicinity of the optic nerve head (ONH).

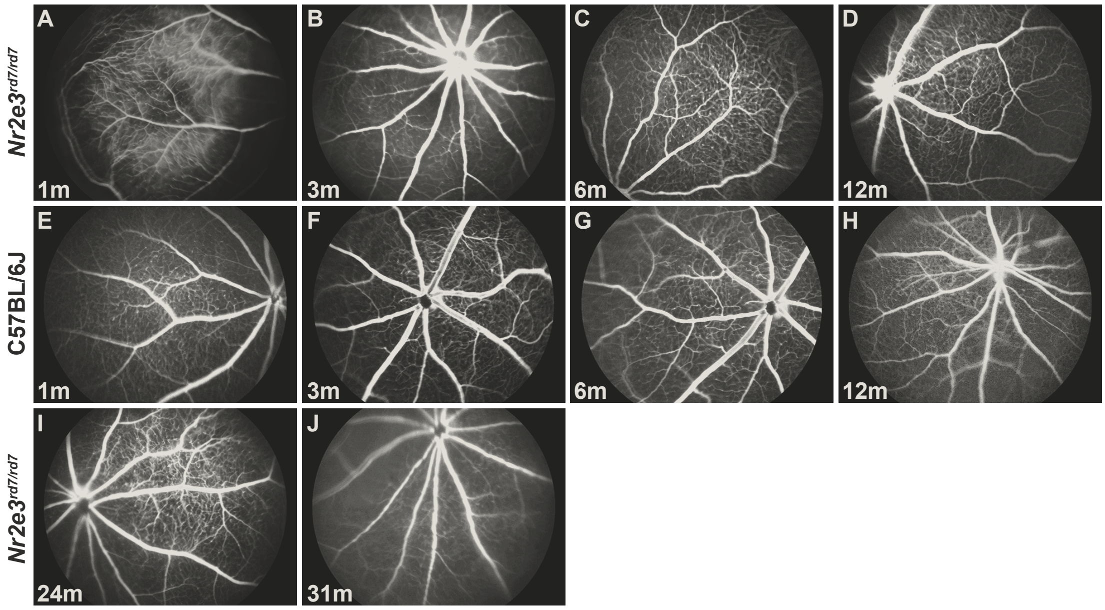

***Supplementary Figure S3*: Fluorescein angiography on C57BL/6J *Nr2e3$^{rd7/rd7}$* retinas.** Retinal vasculature is essentially normal in C57BL/6J *Nr2e3$^{rd7/rd7}$* mice as assessed by fluorescein angiography on C57BL/6J *Nr2e3$^{rd7/rd7}$* retinas at 1 (A), 3 (B), 6 (C), 12 (D), 24 (I) and 31 (J) months (m). As a control, fluorescein angiography was also performed on C57BL/6J retinas at 1 (E), 3 (F), 6 (G) and 12 (H) months.
